# Complete Genome Sequence of Proteus mirabilis Phage Myduc

**DOI:** 10.1128/MRA.01313-19

**Published:** 2019-11-21

**Authors:** Jennifer Tran, Lauren Lessor, Chandler O’Leary, Jason Gill, Mei Liu

**Affiliations:** a Center for Phage Technology, Texas A&M University, College Station, Texas, USA; DOE Joint Genome Institute

## Abstract

Proteus mirabilis as a nosocomial pathogen is often the cause of urinary tract infections. This announcement describes the complete genome sequence of a P. mirabilis myophage named Myduc. Phage Myduc is related to *Enterobacteria* phage phiEcoM-GJ1, which belongs to a group of myophages with small genome sizes (52,000 to 56,000 bp) possessing a T7-like RNA polymerase.

## ANNOUNCEMENT

Proteus mirabilis is a ubiquitous Gram-negative bacterium found in the environment and the human gastrointestinal tract (1). As a leading cause of urinary tract infections, P. mirabilis accounts for a significant portion of all nosocomial infections in the United States (1, 2). As more antibiotic-resistant strains of P. mirabilis are isolated (3), phage therapy may be used in treating infections caused by this pathogen (4).

Phage Myduc was isolated from a wastewater sample collected from College Station, TX, in 2013 using a deidentified Proteus mirabilis clinical isolate as the host. Host bacteria were cultured on nutrient broth or agar (Difco) at 37°C with aeration. Phages were cultured and propagated using the soft agar overlay method (5). The phage was identified as a myophage using negative-stain transmission electron microscopy performed at the Texas A&M University Microscopy and Imaging Center, as described previously (6). Phage genomic DNA was prepared using a modified Promega Wizard DNA cleanup kit protocol, as described previously (6). Pooled indexed DNA libraries were prepared using the Illumina TruSeq Nano low-throughput (LT) kit, and short reads were obtained from the Illumina MiSeq platform using the MiSeq V2 500-cycle reagent kit, following the manufacturer’s instructions, producing 1,296,046 paired-end 250-bp reads for the index containing the phage Myduc genome. FastQC 0.11.5 (https://www.bioinformatics.babraham.ac.uk/projects/fastqc/) was used to quality control the reads. The reads were trimmed with the FASTX-Toolkit 0.0.14 (http://hannonlab.cshl.edu/fastx_toolkit/download.html) before being assembled using SPAdes 3.5.0 (7). Contig completion was confirmed with PCR using primers (5′-TGTTATAGCATCTCTACGAACG-3′ and 5′-GAGAACACTTTGATGCCTAATG-3′) facing off the ends of the assembled contig and Sanger sequencing of the resulting product, with the contig sequence manually corrected to match the resulting Sanger sequencing read. GLIMMER 3.0 (8) and MetaGeneAnnotator 1.0 (9) were used to predict protein-coding genes with manual verification, and tRNA genes were predicted using ARAGORN 2.36 (10). Rho-independent terminators were identified via TransTermHP 2.09 (http://transterm.cbcb.umd.edu/). Sequence similarity searches were performed using BLASTp 2.2.28 (11) with a maximum expectation cutoff of 0.001 against the NCBI nonredundant (nr), UniProt Swiss-Prot (12), and TrEMBL databases. InterProScan 5.15-54.0 (13), LipoP (14), and TMHMM 2.0 (15) were used to predict protein function. All analyses were conducted at default settings via the CPT Galaxy (16) and Web Apollo (17) interfaces (https://cpt.tamu.edu/galaxy-pub).

Phage Myduc has a complete genome of 53,392 bp assembled at 43-fold coverage and a GC content of 39%. The Myduc genome was opened at the position of the direct terminal repeat (3,202 bp long) predicted by PhageTerm (18). Located within the terminal repeat region, a T7-like RNA polymerase gene is the first gene in the Myduc genome. This feature and the presence of all Myduc genes on the top strand suggest that phage Myduc may eject its DNA in a manner similar to that of phage T7, which is assisted by the polymerase’s transcriptional activity. Myduc shares similar genome features and shares at least 44 of its 79 total proteins (BLASTp; E value, <0.001) with a group of small myophages (52,000 to 56,000 bp in genome size) that includes *Enterobacteria* phage phiEcoM-GJ1 (GenBank accession number EF460875) (19), *Escherichia* phages Mangalitsa (GenBank accession number MN045229) (20) and ST32 (GenBank accession number MF044458) (21), and *Pectobacterium* phages PP101 (GenBank accession number KY087898) and PM1 (GenBank accession number KF534715) (22).

### Data availability.

The genome sequence of phage Myduc has been submitted to GenBank under accession number MN098326. The associated BioProject, SRA, and BioSample accession numbers are PRJNA222858, SRR8772108, and SAMN11236500, respectively.
